# From Waste to Bioactive Ingredient: Integrated Extraction, Identification, and Validation of Novel Antioxidant Peptides from Xuefeng Black-Bone Chicken Bones

**DOI:** 10.3390/foods15050942

**Published:** 2026-03-07

**Authors:** Haige Yang, Fanjia Kong, Lan Mo, Yanyang Wu, Aihua Lou, Qingwu Shen, Wei Quan, Lei Zhou, Meichun Li, Yan Liu

**Affiliations:** 1 College of Food Science and Technology, Hunan Agricultural University, Changsha 410128, China; 15043816798@163.com (H.Y.);; 2 Hunan Yunfeifeng Agricultural Company Limited, Huaihua 418200, China

**Keywords:** Xuefeng black-bone chicken bones, antioxidant peptides, enzymatic hydrolysis, response surface methodology, peptide identification, molecular docking

## Abstract

The valorization of poultry bone by-products into high-value bioactive ingredients aligns with the principles of a sustainable circular bioeconomy. This study established an integrated process for the production, identification, and validation of bioactive antioxidant peptides from Xuefeng black-bone chicken bones (BCB). Alcalase was selected as the optimal protease due to its superior performance in both the degree of hydrolysis and antioxidant activity under the optimized conditions. Using response surface methodology (RSM), the optimal hydrolysis conditions were determined as 50 °C, pH 10.18, and 4.2 h, resulting in a hydrolysate with a hydrolysis degree of 25.10% and ABTS radical scavenging activity of 84.36%. Upon ultrafiltration, the <3 kDa fraction demonstrated a significantly higher antioxidant capacity than the crude hydrolysate. Further purification through gel filtration chromatography yielded the F3 sub-fraction (predominantly <1 kDa peptides), which exhibited the most potent activity across all four antioxidant assays conducted (ABTS, DPPH, hydroxyl radical scavenging, and reducing power). A liquid chromatography–tandem mass spectrometry (LC-MS/MS) analysis of F3 led to the identification of 21 peptide sequences. An in silico screening based on bioactivity and toxicity predictions pinpointed three promising candidates: DYPF, WDY, and FGYK. These peptides were chemically synthesized and validated to possess significant in vitro radical scavenging activities against both DPPH and hydroxyl radicals. Molecular docking simulations revealed that all three peptides could spontaneously bind to the Keap1 protein with a high affinity (binding energy < −7.0 kcal/mol), primarily through hydrogen bonds and hydrophobic interactions, suggesting a possible molecular mechanism that may involve the Keap1-Nrf2-ARE antioxidant pathway. This computational insight provides a testable hypothesis for their bioactivity, the verification of which is contingent upon future studies demonstrating their cellular delivery and intracellular action. This work not only provides a sustainable strategy for BCB utilization but also identifies potent antioxidant peptides with potential applications in functional foods and nutraceuticals.

## 1. Introduction

The global poultry industry generates substantial quantities of bone by-products, often regarded as low-value waste, leading to significant economic and environmental challenges. The effective valorization of these by-products is crucial for advancing sustainable food systems and improving the circular economy within the agri-food sector. Chicken bones, rich in proteins [1], particularly collagen, represent a promising but underexploited source for bioactive hydrolysates and peptides. Enzymatic hydrolysis is a preferred, environmentally benign method of unlocking these embedded bioactivities, transforming structural proteins into functional peptides with potential health benefits.

Antioxidant peptides have emerged as a novel class of safe and naturally sourced antioxidants [2]. The extraction of bioactive compounds with antioxidant properties from natural foods has become a key research focus in food science [3]. Peptides sourced from animals and plants have gained significant interest for their sustainable production, economic viability, and favorable safety profiles [4]. In recent years, a wide range of hydrolyzed proteins from plants and animals have been identified as possessing significant biological activities. These include peptides from fish [5,6], animal bones [7,8], and grains [9]. Studies have demonstrated that antioxidant peptides can serve as effective natural alternatives to synthetic antioxidants and hold potential for applications in functional food formulations and health-related fields [10].

The Xuefeng black-bone chicken, a prized indigenous breed in China, is renowned for its unique nutritional and medicinal properties, attributed to its high melanin and other bioactive compound content [11,12]. While its meat is highly valued, the bones, a major by-product of processing, are largely discarded or underutilized. Preliminary studies suggest that black-bone chicken tissues may possess a superior antioxidant potential compared to regular breeds, implying that their bones could be a uniquely potent source of antioxidant peptides [13]. However, systematic research focused on the enzymatic conversion of Xuefeng black-bone chicken bones into bioactive peptides, particularly targeting the identification of specific peptide sequences responsible for antioxidant activity, remains strikingly absent.

To bridge this knowledge gap and realize the untapped potential of this by-product, the present study was designed with a comprehensive strategy. The primary objective is to convert Xuefeng black-bone chicken bones into high-value antioxidant peptides through an integrated approach. This involves, firstly, the systematic optimization of the enzymatic hydrolysis process using response surface methodology to maximize both the hydrolysis efficiency and the resultant antioxidant activity of the hydrolysate. Subsequently, the active components will be isolated and purified through a multi-step chromatographic protocol, leading to the identification of the predominant peptide sequences via LC-MS/MS. Finally, to unequivocally confirm the source of bioactivity, the identified peptides will be chemically synthesized, and their in vitro antioxidant efficacy rigorously will be validated using established assays. The significance of this work is threefold, directly stemming from its methodological design. Firstly, it offers a tangible solution for the sustainable valorization of poultry processing waste, aligning with circular bioeconomy principles by transforming an environmental liability into a valuable resource. Scientifically, it contributes fundamental knowledge to the field of food-derived bioactive peptides by elucidating novel sequences from a unique source, thereby enriching the understanding of structure–activity relationships specific to avian collagen peptides. Practically, the identified and validated antioxidant peptides hold immediate promise as natural, functional ingredients for the development of health-promoting foods, nutraceuticals, and cosmetic products, ultimately enhancing the economic viability and product spectrum of the Xuefeng black-bone chicken industry.

## 2. Materials and Methods

### 2.1. Materials

Fresh Xuefeng black-bone chicken bones (BCB) were supplied by Hunan Yunfeifeng Agricultural Co., Ltd. (Hongjing, China). The following enzymes and reagents were purchased from Shanghai Yuanye Bio-Technology Co., Ltd. (Shanghai, China): Alcalase protease (S10154, 200 U/mg), neutral protease (S10013, 100 U/mg), papain (S10011, 800 U/mg), Flavourzyme proteinase (S10153, 20 U/mg), bromelain (S10009, 300 U/mg), compoundproteinase (S10153, 120 U/mg), 2,2-azino-bis(3-ethylbenzothiazoline-6-sulfonic acid) (ABTS), and 1,1-diphenyl-2-picrylhydrazyl (DPPH). All other chemicals used were of analytical grade.

### 2.2. Preparation of BCB Hydrolysate

BCB with non-bone tissues removed was combined with water at a 1:2 (*w*/*v*) ratio and sterilized using high-temperature, high-pressure steam (125 °C, 30 min). After cooling, the upper layer of oil was removed. The resulting material was dried at 50 °C for 16 h, cooled to room temperature, and subsequently ground using a high-speed grinder.

The BCB powder, after being passed through a 60-mesh sieve, was dispersed in distilled water at a solid-to-liquid ratio of 1:20 (*w*/*v*). The suspension was then hydrolyzed separately using six enzymes (Alcalase, neutral protease, bromelain, compound proteinase, Flavourzyme, and papain). The enzyme-to-substrate ratio was 1:20 (*v*/*w*, enzyme solution volume to BCB powder weight). Prior to use, the activity of each enzyme solution was adjusted to 100 U/mL. The pH of the mixture was adjusted to the target value for each enzyme using 0.1 M NaOH or HCl solutions. The enzymatic hydrolysis was conducted under the respective optimal conditions for each enzyme: Alcalase protease (pH 10, 50 °C), neutral protease (pH 7, 45 °C), bromelain (pH 6.5, 55 °C), compound proteinase (pH 7.5, 50 °C), Flavourzyme proteinase (pH 7.0, 50 °C), and papain (pH 6, 55 °C). After enzymatic hydrolysis, the solutions were heated and maintained at 85 °C for 15 min to inactivate the enzymes completely. The hydrolysates were then centrifuged at 5000× *g* for 8 min at 4 °C. The peptide concentration of each hydrolysate was determined using a bicinchoninic acid (BCA) protein assay kit (Solarbio, Beijing, China) according to the manufacturer’s instructions, with bovine serum albumin as the standard. The degree of hydrolysis (DH) was determined using the pH-stat method by monitoring the volume of sodium hydroxide (NaOH) consumed to maintain a constant pH during the enzymatic reaction. The DH was calculated according to Equation (1), as defined by Adler-Nissen [14].

(1) DH(%) = (B × Nb/α × Mp × htot) × 100

where htot is the total number of peptide bonds per gram of BCB protein (7.6 mM/g protein) [15], B is the volume of NaOH (mL) solution used, Nb is the normality of the NaOH (1 M), MP is the protein mass (g) in the sample used, and α is the average dissociation degree of the terminal amino peptides, which was calculated using Equation (2).

(2) α = 10^pH − pK^/1 + 10^pH − pK^

where pH and pK are the values at which the enzymolysis of protein was conducted.

### 2.3. Optimization of Enzymatic Hydrolysis Conditions

Based on the screening results of the optimal protease, single-factor experiments were subsequently performed under fixed conditions including a solid-to-liquid ratio of 1:20 and an enzyme dosage of 8000 U/g. The effects of key hydrolysis parameters, namely pH (8, 9, 10, 11, 12), time (1, 2, 3, 4, 5 h), and temperature (35, 40, 45, 50, 55 °C), were evaluated on the degree of hydrolysis of black-bone chicken bone enzymatic hydrolysate, as well as on ABTS and hydroxyl radical scavenging activities. The aim is to provide reference data for the subsequent response surface methodology (RSM) experimental design.

### 2.4. Response Surface Methodology Design

Based on the outcomes of single-factor experiments, three key parameters, pH (9, 10, 11), hydrolysis time (3 h, 4 h, 5 h), and temperature (45 °C, 50 °C, 55 °C), were selected as independent variables, while maintaining a constant enzyme dosage of 8000 U/g and a solid-to-liquid ratio of 1:20. A three-factor, three-level Box–Behnken experimental design was implemented using Design-Expert 13 software. The degree of hydrolysis and ABTS radical scavenging activity were used as response values. Each experimental group was performed in triplicate under stable conditions.

### 2.5. Antioxidant Activity Measurements

The antioxidant capacity of the black-bone chicken bones hydrolysates was evaluated using a panel of standard assays. Given their hydrophilic nature, the ABTS radical scavenging assay served as the primary method. The DPPH radical scavenging assay was also employed as a benchmark for cross-study comparison, despite its known bias toward more lipophilic systems. Additionally, hydroxyl radical scavenging and reducing power assays were conducted to assess the ability to quench a physiologically relevant radical and the general electron-donating capacity, respectively. Ascorbic acid (vitamin C) was used as a positive control in all assays.

The ABTS radical scavenging activity was determined according to the method described by [16] with appropriate modifications. Briefly, 1 mL of the sample solution was mixed with 1.5 mL of freshly prepared ABTS working solution. The mixture was allowed to react in the dark at room temperature for 6 min, after which the absorbance at 734 nm was immediately measured using a spectrophotometer.

(3) ABTS radical scavenging activity (%) = [(A2 − A1)/A2] × 100

where A_1_ and A_2_ represent the absorbance of the sample and blank, respectively.

Hydroxyl radical scavenging activity was assayed following a reported method [17] with modifications. In brief, 2 mL of sample was combined with 2 mL each of FeSO_4_ (6 mmol/L), H_2_O_2_ (6 mmol/L), and salicylic acid (6 mmol/L), incubated at 37 °C for 30 min, and the absorbance was subsequently recorded at 510 nm.

(4) Hydroxyl radical scavenging activity (%) = [(A1 − A2)/A3] × 100

where A_1_, A_2_, and A_3_ represent the absorbance of the sample, control, and blank groups, respectively.

The DPPH radical scavenging activity was determined according to a previously reported method [17] with slight modifications. Briefly, 1.5 mL of the sample solution was mixed with 1.5 mL of 0.1 mmol/L DPPH in 95% (*v*/*v*) ethanol. The mixture was incubated in the dark at room temperature for 30 min, after which the absorbance was measured at 517 nm using a spectrophotometer.

(5) DPPH radical scavenging activity (%) = [(A1 − A2)/A3] × 100

where A_1_, A_2_, and A_3_ represent the absorbance of the sample, control, and blank groups, respectively.

The reducing power was determined according to the method of [17] with slight modifications. Briefly, 1 mL of the sample solution at varying concentrations was mixed with 2.5 mL of 0.2 M phosphate buffer (pH 6.6) and 2.5 mL of 1% (*w*/*v*) potassium ferricyanide. The mixture was incubated in a water bath at 50 °C for 20 min. After incubation, 2.5 mL of 10% (*w*/*v*) trichloroacetic acid was added, followed by centrifugation at 5000 *g* for 10 min. Subsequently, 2 mL of the supernatant was collected and mixed with 2 mL of distilled water and 0.4 mL of 0.1% (*w*/*v*) ferric chloride solution. The absorbance of the final mixture was measured at 700 nm using a spectrophotometer.

### 2.6. BCB Peptide Separation via Ultrafiltration

The separated components were divided into three parts with molecular weights of <3 kDa, 3–10 kDa and >10 kDa, respectively, using ultrafiltration centrifuge tubes with molecular weight cut-off values of 3 kDa and 10 kDa. Each component was collected and freeze-dried at −20 °C. Subsequently, the antioxidant activity of each component was determined.

### 2.7. Gel Filtration Chromatography for Purification of BCB Peptide

The freeze-dried sample with the highest antioxidant activity (50 mg/mL), obtained following ultrafiltration, was reconstituted in 4 mL of ultrapure water and passed through a 0.45 μm membrane filter. This solution was subsequently loaded onto a Sephadex G-15 gel column (16 mm × 100 cm) pre-equilibrated with ultrapure water. The column was then eluted further using ultrapure water. Fractions of 2 mL per tube were collected, and absorbance was measured at 280 nm using a microplate reader. The collected fractions were subsequently freeze-dried and prepared as a solution (5 mg/mL) for the further assessment of antioxidant activity.

### 2.8. Identification of BCB Peptides Using Liquid Chromatography–Tandem Mass Spectrometry

The LC-MS/MS identification was carried out by referring to the method of [18] with slight modifications. We selected the peptide segment with the highest antioxidant activity in the detachable component. Chromatographic separation was performed using the Easy-nLC 1200 system Thermo Fisher Scientific (Waltham, MA, USA), followed by peptide separation. Then, mass spectrometry analysis was performed using the Q Exactive TM Hybrid Quadrupole-OrbitrapTM Mass Spectrometer (Thermo Fisher Scientific, Waltham, MA, USA). The analytical column (Reprosil-Pur 120 C18-AQ, 150 μm × 17 cm, 1.9 μm, (Thermo Fisher Scientific)) chromatographic conditions are as follows: mobile phase: A: 0.1% formic acid aqueous solution; B: 20% 0.1% formic acid water −80% acetonitrile; total flow rate: 600 nL/min; LC linear gradient: 4% to 8% B, 2 min; 8%~28% B, 33 min; 28% to 40% B, 20 min; 40% to 95% B, 1 min; 95% to 95% B, 10 min.

The MS conditions were established as follows: a scanning range of 100–1500 *m*/*z*; a primary MS resolution of 70,000 with an automatic gain control (AGC) target of 3 × 10^6^ and a maximum injection time (IT) of 100 ms; a secondary MS resolution of 17,500, with an AGC target of 1 × 10^5^ and a maximum IT of 50 ms. Secondary MS activation was performed via higher-energy collisional dissociation (HCD) at a normalized collision energy of 28%. A non-specific enzyme setting and a false discovery rate (FDR) cut-off of 0.01 were applied. The raw MS data were processed and analyzed in PEAKS Studio 11 using the following parameters: a fixed modification of Carbamidomethyl (C); variable modifications including oxidation (M) and acetyl (peptide N-term); a peptide mass tolerance of 20 ppm; a fragment mass tolerance of 0.02 Da; with peptide-spectrum matching performed against the UniProt proteome database (accession: UP000000539).

### 2.9. Computer Virtual Screening of BCB Peptides

The Peptide Ranker server (http://distilldeep.ucd.ie/PeptideRanker/) predicted the probability of identification of peptides with biological activity; in order to reduce the occurrence of a false positive, it chose a peptide score of more than 0.7. The ToxinPred server (http://crdd.osdd.net/raghava/toxinpred/) predicted the potential toxicity of peptides, choosing avirulent peptides. The BIOPEP database (https://biochemia.uwm.edu.pl/biopep-uwm/) was applied to determine the polypeptide novelty and predict the potential biological activity. Unreported and potentially antioxidant activity was determined using the PreDraw tool (http://pepdraw.com/) and the Innovagen tool. Peptide screening was performed through software (http://www.innovagen.com/proteomics-tools) prediction of physical and chemical quality, including molecular weight, isoelectric point, charge, hydrophobicity and water solubility. Finally, an AnOxPePred server (https://services.healthtech.dtu.dk/services/AnOxPePred-1.0/) was used to predict the selected peptide antioxidant activity, giving the predicted free radical scavenger capacity (PFRS), all UR accessed on 5–10 October 2025.

### 2.10. Peptide Synthesis

The selected peptide segments were synthesized through a solid-phase synthesis method from Nanjing Chen tai Biotechnology Co., Ltd. (Nanjing, China) with a purity of over 95%. The synthetic polypeptide should be −20 °C for future use in subsequent antioxidants.

### 2.11. Molecular Docking

In silico molecular docking simulations were conducted to investigate the potential protective mechanisms of antioxidant peptides. Following a modified protocol based on reference [19], the two-dimensional structures of the peptides were constructed using ChemBioDraw Ultra 20.0 and subsequently subjected to energy minimization in ChemBio3D with a Minimum RMS Gradient set to 0.001. The optimized peptide models were then converted into mol2 format. For receptor preparation, the crystal structure of Keap1 (PDB ID: 2FLU) was retrieved from the Protein Data Bank (PDB) and processed using PyMOL 3.0.4, involving the removal of water molecules and non-essential residues, followed by hydrogen addition, before being exported as a pdbqt file. Molecular docking was performed using AutoDock 4.0, with the docking grid center defined at coordinates (x: 12.066, y: 16.085, z: 8.317). The exhaustiveness parameter was maintained at 9. Finally, the docking poses were visualized and analyzed using PyMOL 3.1.0 and LigPlus 1.4.5 software.

### 2.12. Statistical Analysis

Each experiment was performed in triplicate, and the resulting data were expressed as mean ± standard deviation (SD). The data were also subjected to analysis of variance (ANOVA) using SPSS software (SPSS version 27, Chicago, IL, USA). The mean values were compared using Duncan’s post hoc test to determine the statistically significant difference at *p* < 0.05. Design-Expert 13.0 was used to examine the RSM data.

## 3. Results and Discussion

### 3.1. Optimization of Protease for Preparation of BCB Hydrolysate

**Figure 1:**
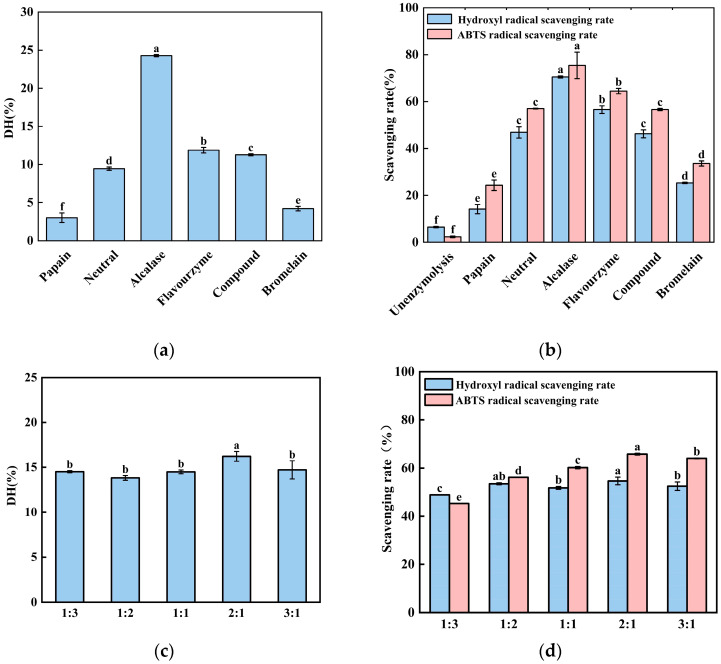
Enzymatic hydrolysis and in vitro antioxidant activity of black-bone chicken bone (BCB) hydrolysates. (**a**) Degree of hydrolysis (DH) of BCB hydrolysates prepared using six single proteases (Alcalase, neutral protease, bromelain, compound protease, Flavourzyme, and papain) at their respective optimal pH and temperature with a total enzyme dosage of 8000 U/g substrate. (**b**) ABTS and hydroxyl radical scavenging activities (measured at 10 mg/mL) of the hydrolysates corresponding to (**a**). (**c**) DH of BCB hydrolysates prepared with combined Alcalase and Flavourzyme, with the total enzyme dosage kept constant at 8000 U/g substrate and the Alcalase-to-Flavourzyme volume ratio varying as indicated on the *x*-axis. (**d**) ABTS and hydroxyl radical scavenging activities (measured at 10 mg/mL) of the hydrolysates corresponding to (**c**). Data are presented as the mean ± standard deviation (SD) (*n* = 3). Within each panel (**a**–**d**), bars topped with different lowercase letters are significantly different (*p* < 0.05), as determined using one-way ANOVA followed by Duncan’s multiple range test. Statistical comparisons were performed independently for the ABTS and hydroxyl radical scavenging data in panels (**b**,**d**).

Enzymatic proteolysis is the predominant method for generating bioactive peptides from protein sources. The degree of hydrolysis serves as a fundamental parameter for quantifying the extent of proteolytic cleavage. The degree of hydrolysis (DH) is a key indicator for measuring the extent of protein decomposition, reflecting the ratio of the number of peptide bonds broken to the total number of peptide bonds in the protein substrate [8]. As shown in Figure 1a, the degree of hydrolysis of black-bone chicken bone meal varied significantly among the six proteases. The degree of hydrolysis (24.27%) in the Alcalase protease-treated group was the highest, followed by the Flavourzyme-treated group (11.88%). Meanwhile, the degree of hydrolysis in the papain-treated group was the lowest (3.02%). The data in Figure 1b showed that the scavenging rates of ABTS (75.46%) or hydroxyl radicals (70.52%) were highest in the alkaline protease-treated group. This implied that the radical scavenging capacity of the hydrolysates also exhibited variation. This disparity may be attributed to the fact that alkaline proteases are endogenous enzymes with a broad substrate specificity, enabling them to cleave a wide range of peptide and amide bonds within protein substrates [20]. This was followed by the Flavourzyme protease hydrolysate-treated group, whilst the papain hydrolysate-treated group showed the lowest radical scavenging capacity. These differences may be attributed to variations in the cleavage specificity among protease types [20].

The hydrolysis effects of Alcalase protease and Flavourzyme protease on black chicken bone powder, as well as the free radical scavenging efficiency of their enzymatic products, are superior to other enzymes. Therefore, a combination of Alcalase protease and Flavourzyme protease were used to treat black chicken bone powder. This was carried out under the following fixed conditions in our experiments: a total enzyme dosage of 8000 U/g, a material-to-liquid ratio of 1:20, a temperature of 50 °C, and a pH of 8. Alcalase protease and Flavourzyme protease were mixed at five different (*v*/*v*) ratios: 1:3, 1:2, 1:1, 2:1, and 3:1. As shown in Figure 1c, at a ratio of 2:1, the highest degree of hydrolysis was achieved. Our data also indicated that the radical scavenging rate of the enzymatic hydrolysates was also lower than that of the hydrolysates from the Alcalase protease-treated group. This reduction in performance may be attributed to the differing optimal pH and temperature ranges of Alcalase protease and Flavourzyme protease, making it difficult to satisfy the ideal reaction conditions for both enzymes simultaneously in a single hydrolysis system. Our data showed that neither the hydrolysis efficiency nor the bioactivity reached the level achieved by the treatment of alkaline protease. So, the Alcalase protease was selected for subsequent experiments.

### 3.2. Optimization of Hydrolysis Conditions Using Single-Factor Tests

**Figure 2:**
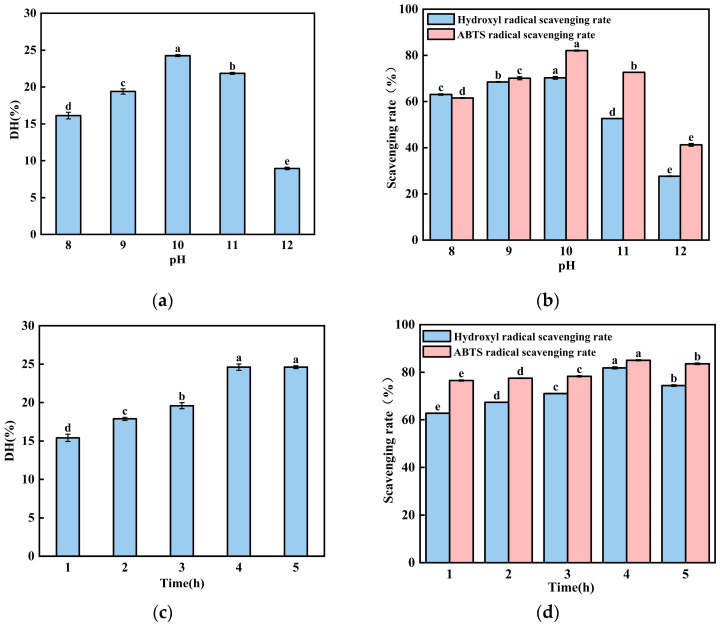

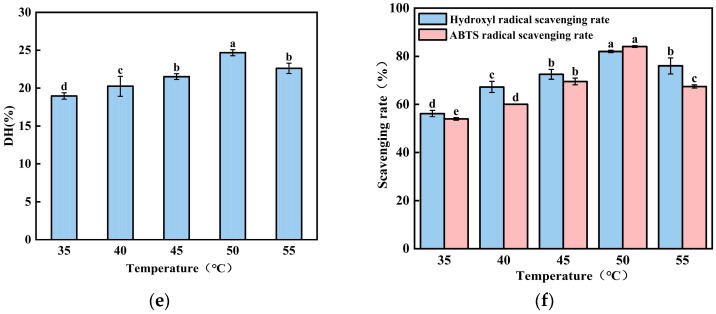
The effects of three key extraction parameters, pH (**a**,**b**), enzymatic time (**c**,**d**), and enzymatic temperature (**e**,**f**), on the degree of hydrolysis (**a**,**c**,**e**) and on the antioxidant activity (**b**,**d**,**f**) of the resulting protein hydrolysates are shown. Antioxidant activity was evaluated by measuring the scavenging rates against both ABTS radicals and hydroxyl radicals. Data points represent the mean ± standard deviation of three independent replicates. Within each panel, means labeled with the same lowercase letter are not significantly different, while those with different letters indicate statistically significant differences (*p* < 0.05).

The value of pH plays a critical role in hydrolysis efficiency. It directly influences enzyme activation and modulates the binding and dissociation dynamics between the enzyme and substrate [21,22]. As shown in Figure 2a,b, the degree of hydrolysis and radical scavenging rate of the enzymatic products exhibited an upward trend at pH 8.0 and reached its maximum value in the pH 10-treated group. Meanwhile, it gradually decreased in the pH 10- and 12-treated groups. According to these data, we can conclude that the radical scavenging rate shows a consistent trend with the changes in the degree of hydrolysis under the treatment of the same value of pH. It suggests that pH influences the dissociation state of amino and carboxyl groups on the protease molecule. Conversely, at higher pH values, excess alkalinity may disrupt the structure of enzymes and hinder the enzymatic reaction [23]. Therefore, the optimal pH value is 10.

Hydrolytic time plays a crucial role in hydrolysis efficiency [24]. As shown in Figure 2c,d, the degree of hydrolysis and the radical scavenging rate of the enzymatic hydrolysates increased significantly with the extension of the reaction time. It reached its maximum value in the 4 h treatment group. Our data imply that an insufficient hydrolysis time prevents the proteins from being hydrolyzed into peptides. If the enzymatic hydrolysis time is too long, it can lead to an excessive digestion of proteins, producing more free amino acids [25,26]. So, 4-h treatment was selected for subsequent experiments.

The hydrolysis temperature is a significant factor influencing the yield of soluble peptides. As shown in Figure 2e, when the hydrolysis temperature increased from 35 to 50 °C, the degree of hydrolysis and the radical scavenging capacity of the enzymatic products from black-bone chicken bone meal gradually increased with the Alcalase protease, reaching a peak at 50 °C. However, when the temperatures exceeded 50 °C, both the hydrolysis degree of the black-bone chicken bone meal and the radical scavenging capacity of the enzymatic products exhibited a declining trend. This occurs because, within the optimal temperature range, elevated temperatures increase the internal energy of the enzyme molecules, thereby enhancing the efficiency of antioxidant peptide production. Conversely, excessively high temperatures may alter the structure of the enzyme’s active site, preventing substrate binding and thereby inhibiting the catalytic reaction [15].

Single-factor experiments indicated that pH, hydrolytic time and temperature had a significant impact on the DH and antioxidant capacity of BCB; pH (9, 10, 11), hydrolytic time (3 h, 4 h, 5 h), and hydrolysis temperature (45 °C, 50 °C, 55 °C) were chosen for subsequent experiments.

### 3.3. Optimization of BCB Enzymatic Hydrolysis Process by Response Surface Methodology

A response surface methodology was applied to further enhance the BCB hydrolysis process; DH and ABTS radical scavenging activity were selected as the key response variables to evaluate the efficiency and bioactivity of the enzymatic hydrolysates. The experimental design matrix along with the corresponding results for DH and ABTS activity are presented in Table 1. The data revealed a considerable variation in both DH (ranging from 16.97% to 25.70%) and ABTS scavenging activity (from 63.09% to 84.81%) across the different combinations of temperature (A), time (B), and pH (C). The central point experiments (Groups 5, 8, 9, 11, 17) demonstrated a high reproducibility and yielded the most favorable outcomes, with the highest observed values reaching 25.70% for DH and 84.81% for ABTS activity under the conditions of 50 °C, 4 h, and pH 10. This preliminary indication pointed towards these conditions as a potential optimum within the studied range.

Subsequently, the mathematical model of the data in the table was established, and variance analysis was conducted using Design-Expert 13 software. The quadratic regression model constructed was

(6) DH = 24.89 + 0.8139A + 0.3111B + 1.30C − 0.3583AB − 1.42AC + 0.5404BC − 1.32A^2^ − 0.5846B^2^ − 3.10C^2^

(7) ABTS radical scavenging rate = 84.23 + 0.9137A + 1.25B + 2.16C − 0.1459AB − 1.52AC + 1.05BC − 8.22A^2^ − 3.93B^2^ − 7.25C^2^

The analysis of variance (ANOVA) for the DH model is summarized in Table 2. The model was highly significant (*p* < 0.0001), with an F-value of 32.68, and the lack of fit was non-significant (*p* = 0.3150), confirming the model’s adequacy. The coefficient of determination (R^2^ = 0.9768) and adjusted R^2^ (0.9469) indicated that the model could explain over 97% of the variation in DH. Among the linear terms, temperature (A, *p* = 0.0033) and pH (C, *p* = 0.0002) exerted extremely significant positive effects on DH. The quadratic terms of A^2^ (*p* = 0.0013) and C^2^ (*p* < 0.0001) were also highly significant, suggesting a curvilinear relationship between these factors and DH. Furthermore, the interaction between temperature and pH (AC, *p* = 0.0010) was significant, implying that the effect of pH on DH is dependent on the hydrolysis temperature. The ANOVA for the model of ABTS radical scavenging activity is presented in Table 3. This model was also highly significant (*p* < 0.0001, F-value = 62.44), with a non-significant lack of fit (*p* = 0.0728). The high R^2^ (0.9877) and adjusted R^2^ (0.9719) values demonstrated an excellent fit. For the antioxidant activity, pH (C) was the most significant linear factor (*p* = 0.0009), followed by hydrolysis time (B, *p* = 0.0161). Notably, all quadratic terms (A^2^, B^2^, C^2^) were extremely significant (*p* < 0.0001 for A^2^ and C^2^; *p* = 0.0002 for B^2^), indicating that the ABTS activity response surface has a strong curvature. The interaction between temperature and pH (AC, *p* = 0.0299) was a significant interactive term, highlighting the combined influence of these two parameters on the release of antioxidant peptides.

**Figure 3:**
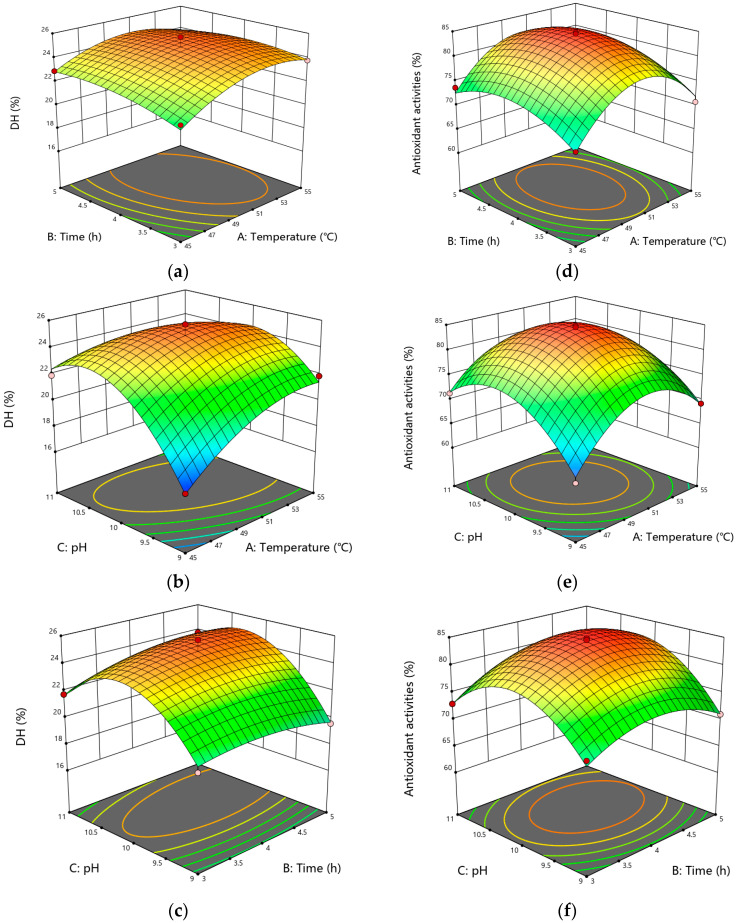
Response surface plots showing correlation of (**a**) hydrolysis time and temperature; (**b**) hydrolysis pH and temperature; (**c**) hydrolysis pH and time on the DH of black-bone chicken bones. (**d**) Hydrolysis time and temperature; (**e**) hydrolysis pH and temperature; (**f**) hydrolysis pH and time on the ABTS radical scavenging activities of black-bone chicken bones hydrolysates.

The interaction between different factors was visualized as a response surface plot in Figure 3. The interaction between temperature and pH had marked impacts on DH, and the interaction of temperature and pH markedly influenced the ABTS radical scavenging activity. The steepness of the 3D response surface plots indicated that pH exerted the most substantial effect on DH, followed by temperature, with the time exhibiting the least influence, which was consistent with the ANOVA results of the response surface.

In summary, the RSM analysis successfully established statistically robust models for both hydrolysis efficiency (DH) and bioactivity (ABTS). The results identified pH as a critically influential factor for both responses, while temperature and time played distinct yet important roles. The significant interaction between temperature and pH underscores the complexity of the enzymatic process. The derived models provide a reliable predictive tool for optimizing the hydrolysis of BCB, with the central point conditions (50 °C, 4 h, pH 10) emerging as the provisional optimum based on the experimental data. This optimized protocol was therefore adopted for the large-scale preparation of BCB hydrolysate for subsequent purification steps.

### 3.4. Verification of Predicted Models and Antioxidant Activity Analysis

The optimal enzymatic hydrolysis conditions for BCB, as predicted by the RSM model, were temperature 50.3952 °C, time 4.2223 h, and pH 10.1853. Under these conditions, the model predicted a DH of 25.1151% and an ABTS radical scavenging rate of 84.5066%. For practical experimental validation, the parameters were slightly adjusted to 50 °C, 4.2 h, and pH 10.18, considering equipment precision and operational feasibility. Triplicate verification experiments yielded an average DH of 25.0988 ± 0.61% and an ABTS scavenging rate of 84.3605 ± 0.50%. The close agreement between the experimental results and model predictions confirms the high reliability and practical applicability of the established RSM model for optimizing the enzymatic hydrolysis process of BCB.

**Figure 4:**
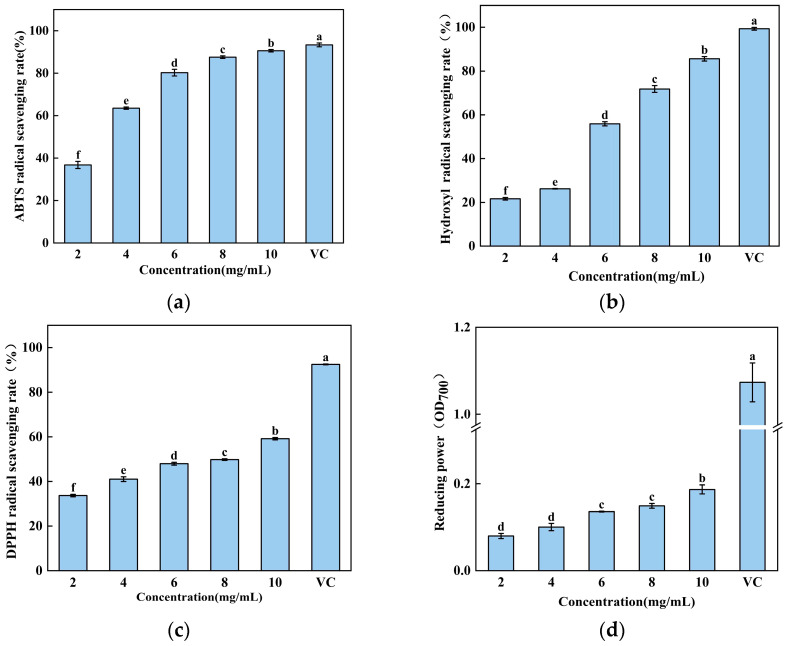
(**a**) ABTS free radical scavenging rate, (**b**) hydroxyl free radical scavenging rate, (**c**) DPPH free radical scavenging rate and (**d**) reducing power of BCP at different concentrations were determined separately, with ascorbic acid (VC) at a concentration of 10 mg/mL used as a positive control. The results were expressed as the mean ± standard deviation of three determinations. Different lowercase letters indicate significant differences among different concentrations of hydrolysates (*p* < 0.05).

To further characterize the antioxidant potential of the hydrolysate obtained under these optimized conditions, its in vitro activity was evaluated using a multi-assay approach. As shown in Figure 4, the hydrolysate exhibited strong, concentration-dependent antioxidant effects across all tested systems. The ABTS radical scavenging activity increased sharply from 36.77 ± 1.36% at 2 mg/mL to 90.59 ± 0.47% at 10 mg/mL. Similarly, the hydroxyl radical scavenging capacity demonstrated a pronounced dose–response, rising from 21.67 ± 0.48% to 85.61 ± 0.81% over the same concentration range. The DPPH radical scavenging activity and ferric reducing power also showed consistent increases, progressing from 33.68 ± 0.44% to 59.16 ± 0.43% and from an OD700 of 0.079 to 0.187, respectively.

Collectively, these results accomplish two key objectives: firstly, they validate the accuracy of the RSM model in predicting optimal hydrolysis conditions for maximizing both hydrolysis efficiency and bioactivity; secondly, they comprehensively demonstrate that the resulting BCB hydrolysate possesses potent and broad-spectrum in vitro antioxidant properties. The concentration-dependent efficacy across multiple mechanistic assays—including electron transfer (ABTS, DPPH, reducing power) and the highly reactive radical neutralization hydroxyl free radical scavenging rate—indicates the presence of a complex mixture of bioactive peptides capable of acting through diverse antioxidant pathways. This confirms that the optimized enzymatic process successfully converts BCB into a functionally valuable hydrolysate with significant potential for use as a natural antioxidant ingredient.

### 3.5. Separation and Antioxidant Activity of BCB-Derived Peptides

**Figure 5:**
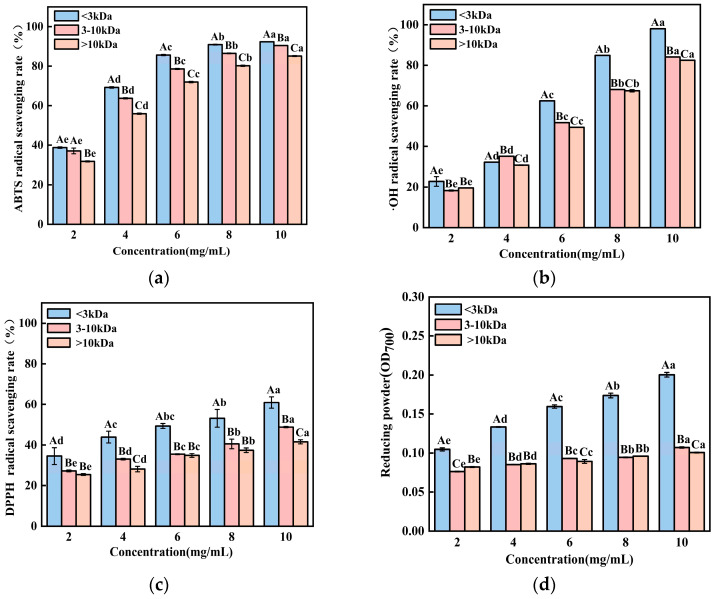
(**a**) ABTS radical scavenging rate, (**b**) hydroxyl radical scavenging rate, (**c**) DPPH radical scavenging rate, and (**d**) reducing power of enzymatic hydrolysates from BCB with varying molecular weights. Lowercase letters denote significant differences in antioxidant activity among peptide segments of the same molecular weight but different concentrations (*p* < 0.05). Uppercase letters indicate significant differences among different molecular weights at the same concentration (*p* < 0.05).

Molecular weight is a critical factor influencing the antioxidant capacity of protein hydrolysates, with lower-molecular-weight peptides generally exhibiting a superior activity due to their higher bioavailability and greater exposure of reactive groups [27]. To isolate and characterize the antioxidative components from the BCB hydrolysate, ultrafiltration was employed using membranes with molecular weight cut-offs (MWCO) of 10 kDa and 3 kDa. This process separated the crude hydrolysate into three distinct fractions: >10 kDa, 3–10 kDa, and <3 kDa. As illustrated in Figure 5, all fractions demonstrated concentration-dependent increases in antioxidant activity across the tested assays, exhibiting approximately linear trends within the experimental concentration range. Notably, the <3 kDa fraction consistently displayed the strongest antioxidant performance. At a concentration of 10 mg/mL, this fraction showed an ABTS radical scavenging activity of 92.32 ± 0.04%, hydroxyl radical scavenging of 98.04 ± 0.10%, DPPH radical scavenging of 60.93 ± 2.30%, and a reducing power of 0.20 ± 0.002. Compared to the unfractionated hydrolysate, these values represent increases of 1.9%, 14.5%, 3.0%, and 7.0%, respectively. Notably, the <3 kDa ultrafiltration fraction demonstrated a significant enhancement in antioxidant activity across all evaluated assays relative to the total hydrolysate. This was most pronounced for hydroxyl radical scavenging, where activity increased from 85.61% to 98.04%, indicative of the successful concentration of highly active peptide antioxidants. The enhanced bioactivity of the <3 kDa fraction can be attributed to its smaller peptide size, which provides a larger specific surface area and better exposure of active amino acid residues (e.g., Tyr, Phe, Trp, His, and Cys). These structural features facilitate electron or hydrogen atom transfer to free radicals, thereby improving radical quenching efficiency. The 3–10 kDa fraction retained moderate antioxidant activity, likely due to the presence of some accessible active sites, though its more complex structure may partially hinder interaction with radicals. In contrast, the >10 kDa fraction showed the lowest activity, as its larger and more folded conformation likely encapsulates potential reactive sites, reducing the binding capacity with free radicals. To confirm that the observed bioactivity stemmed from peptides rather than co-extracted low-molecular-weight metabolites, a non-hydrolyzed BCB control was subjected to identical ultrafiltration. The resulting control fractions (3–10 kDa and >10 kDa) exhibited a negligible antioxidant activity across all assays. Although minimal baseline activity was detected in the <3 kDa control fraction, it accounted for less than 10% of the activity in the corresponding enzymatically hydrolyzed <3 kDa fraction (see Supplementary Figure S1). Therefore, these control experiments conclusively demonstrate that the potent antioxidant capacity of the ultrafiltration fractions is overwhelmingly (>90%) attributable to the peptides released during enzymatic hydrolysis. These findings align with previous reports on bioactive peptide fractions from various animal protein hydrolysates. For instance, Yang et al. observed that the <3 kDa fraction from duck plasma hydrolysate possessed a higher antioxidant activity than larger counterparts [8]. Similarly, Zhang et al. and Zhu et al. reported that the lowest molecular weight fractions from respective protein hydrolysates exhibited the strongest antioxidant effects [28,29]. Collectively, these results confirm that enzymatic hydrolysates enriched with low-molecular-weight peptides demonstrate significantly enhanced antioxidant potential, underscoring the effectiveness of ultrafiltration as a preliminary step in concentrating bioactive peptides from BCB hydrolysates.

### 3.6. Further Purification and Antioxidant Evaluation of Ultrafiltered Fractions via Gel Filtration Chromatography

**Figure 6:**
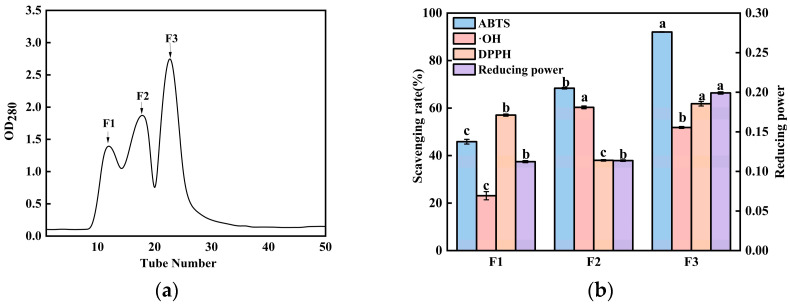
(**a**) Elution profile from size-exclusion chromatography using a Sephadex G-15 column, showing the separation of the <3 kDa BCP into multiple peptide sub-fractions (F1, F2, F3). (**b**) Antioxidant activities of the collected G-15 sub-fractions, each tested at a fixed concentration of 5.0 mg/mL. Activities were evaluated using four independent assays: ABTS radical scavenging, hydroxyl radical scavenging, DPPH radical scavenging, and reducing power. Data are presented as the mean ± standard deviation (*n* = 3). Lowercase letters denote significant differences in antioxidant activity among peptide segments (*p* < 0.05).

To further isolate and identify the most active antioxidant peptides, the <3 kDa ultrafiltration fraction, which demonstrated the highest antioxidant capacity, was subjected to size-exclusion chromatography using a Sephadex G-15 column. This technique separates molecules based on their hydrodynamic volume, with larger molecules eluting earlier than smaller ones [30]. As shown in Figure 6a, three distinct fractions (F1, F2, and F3) were successfully separated, eluting in order of decreasing molecular weight (F1 > F2 > F3).

The antioxidant activities of these three fractions were systematically evaluated and are presented in Figure 6b. At a concentration of 5 mg/mL, the scavenging rates against ABTS and DPPH radicals, as well as the reducing power, followed the order F3 > F2 > F1. Notably, fraction F2 exhibited the highest hydroxyl radical scavenging activity. However, considering a comprehensive analysis across multiple antioxidant indicators, fraction F3 demonstrated the strongest overall antioxidant capacity. This superior activity is consistent with its position as the smallest-molecular-weight fraction obtained from the chromatographic separation. The enhanced antioxidant performance of low-molecular-weight peptides (e.g., F3) can be attributed to their structural characteristics. Smaller peptides typically possess more flexible conformations and a greater exposure of key functional groups (e.g., hydrophobic amino acids and hydrogen donors), enabling a more efficient electron or hydrogen atom transfer to neutralize free radicals. Gel filtration purification not only enriches these low-molecular-weight peptides but also reduces interference from higher-molecular-weight species or non-peptide impurities, thereby rendering the activity of the target components more pronounced.

These findings align with the established literature on antioxidant peptides. Multiple studies have reported that peptides with lower molecular weights generally exhibit a superior antioxidant capacity, whereas larger or aggregated peptides may show a reduced activity due to steric hindrance and the shielding of active sites within their structures. For instance, Sarbon et al. separated chicken breast protein hydrolysate via Sephadex G-25 and found that the lower-molecular-weight fraction possessed strong DPPH radical scavenging and reducing abilities [31]. Similarly, Ouyang et al. reported that smaller walnut peptide fractions outperformed larger ones under oxidative stress [18], and Tang et al. emphasized the close inverse relationship between peptide molecular weight and antioxidant activity [32]. Our results corroborate this prevailing theory, confirming that molecular weight is a key determinant of antioxidant efficacy in peptide mixtures. Based on its consistently high activity across multiple assays, fraction F3 was selected for subsequent structural identification with mass spectrometry.

### 3.7. Identification and Characterization of Antioxidant Peptides from the F3 Fraction Using LC-MS/MS

To identify the specific peptide sequences responsible for the superior antioxidant activity observed in the F3 fraction, the purified sample was subjected to liquid chromatography–tandem mass spectrometry (LC-MS/MS) analysis. The LC-MS/MS analysis of the highly active fraction F3 (isolated by Sephadex G-15 size-exclusion chromatography) identified a total of 6488 peptides. As shown in Table 4, all detected peptides had a molecular weight (MW) below 3 kDa. The distribution was dominated by low-MW species, with 6429 peptides (<1 kDa) accounting for 99.1% of the total. The remaining peptides comprised 57 within the 1–1.5 kDa range and only two within the 1.5–3 kDa range, collectively confirming the predominance of low-MW peptides in this fraction.

The rapid development of bioinformatics enables researchers to predict the biological characteristics of peptides using online databases. The European Food Safety Authority (EFSA) has emphasized the importance of applying bioinformatics databases to predict the biological functions of peptides [33]. The molecular weight is concentrated within the range of 200–1000 kDa. To further derive novel antioxidant peptides with potential activity from the identified peptide sequences, bioinformatics analysis will be conducted on the identified peptides. Following database searching and bioinformatic filtering, a total of 21 novel peptide sequences were successfully identified from the BCB hydrolysate. Their physicochemical properties and predicted bioactivities are summarized in Table 5. As shown in Table 5, first, peptide segments with peak areas greater than 0 were selected to ensure their actual existence, resulting in 6052 peptide segments. Then, we selected −10lgP > 20 (FDR: 1%). The Peptide Ranker website predicts that there are a total of 258 peptide segments with a biological activity score greater than 0.7. Then, through the BIOPEP database, new peptides with potential antioxidant activity and the AnOxPePred-1.0 online tool were selected for antioxidant activity score evaluation. The website predicted that there were a total of 21 peptides with an antioxidant score > 0.5, and all the screened 21 peptides were non-toxic. Finally, the Innovagen tool was used for water solubility prediction, and three peptide segments were obtained, with sequences of DYPF, WDY, and FGYK. The isoelectric points of the three peptide segments are distributed between 2.95 and 9.48, the net charge range is −1 to 1, and the hydrophobicity value is between 8.74 and 9.43. Studies show that the antioxidant activity of polypeptides is related to their molecular weight distribution. Peptides with a molecular weight of 500–800 Da from vertebrates usually have a high antioxidant activity [3]. Next, molecular docking simulations will be conducted on these three peptide segments to further verify their antioxidant activity.

### 3.8. In Vitro Antioxidant Activity of Synthesized Peptides

To conclusively validate the bioactivity of the novel peptides identified from the BCB hydrolysate, the sequences DYPF, WDY, and FGYK were chemically synthesized, and their in vitro antioxidant capacities were experimentally assessed. As summarized in Table 6, all three synthesized peptides demonstrated notable free radical scavenging activity in a dose-dependent manner. At a concentration of 1 mg/mL, the DPPH radical scavenging rates for WDY, DYPF, and FGYK were 28.10 ± 2.57%, 31.06 ± 1.88%, and 33.99 ± 1.55%, respectively. Similarly, their hydroxyl radical scavenging activities were measured at 34.51 ± 0.70%, 36.78 ± 1.12%, and 50.41 ± 0.78%, respectively. Pairwise comparisons confirmed that FGYK displayed a significantly stronger (*p* < 0.05) hydroxyl radical scavenging activity relative to both WDY and DYPF, while its DPPH scavenging capacity was also statistically superior to that of WDY. Collectively, these results demonstrate that, among the peptides examined, FGYK possesses the most pronounced overall antioxidant efficacy.

The antioxidant potency of peptides is often influenced by their specific amino acid composition and sequence. For instance, peptides rich in glycine (G), alanine (A), proline (P), aspartic acid (D), and asparagine (N) have been associated with enhanced radical scavenging ability, a property attributed to the unique chemical properties of these residues [34]. Furthermore, the presence of hydrophobic amino acids (e.g., A, P, V, I, L, F) and the nature of terminal residues are critical determinants of a peptide’s capacity to quench radicals [6]. Additional contributions can arise from residues such as glutamine (Q), arginine (R), serine (S), and threonine (T). The sequences of DYPF, WDY, and FGYK contain combinations of such functional residues—including aromatic (Y, F, W), acidic (D), and basic (K) amino acids—which likely synergize to confer their observed antioxidant activity through mechanisms such as hydrogen donation and electron transfer [35,36,37].

### 3.9. Molecular Docking Simulation for Analyzing the Antioxidant Properties of Identified Peptides

Molecular docking serves as a powerful computational tool in bioactive peptide research, enabling the detailed characterization of peptide–receptor interactions through conformational analysis and binding affinity assessment [38]. To explore the potential molecular mechanism underlying the antioxidant activity of the identified peptides, we performed in silico docking studies targeting the Kelch-like ECH-associated protein 1 (Keap1), a key regulator of the cellular oxidative stress response. The Keap1-Nrf2-ARE signaling pathway is a central defense mechanism against oxidative damage [39]. Under normal conditions, Keap1 binds to and promotes the degradation of the transcription factor Nrf2, suppressing the expression of antioxidant genes. Upon exposure to oxidative stress or interaction with certain bioactive compounds, this binding can be disrupted, allowing Nrf2 to translocate to the nucleus and activate the Antioxidant Response Element (ARE), thereby upregulating a suite of cytoprotective genes [2]. It has been hypothesized that antioxidant peptides may exert their effects by directly binding to Keap1, preventing its interaction with Nrf2 and thus potentiating the cellular antioxidant response [40].

**Figure 7:**
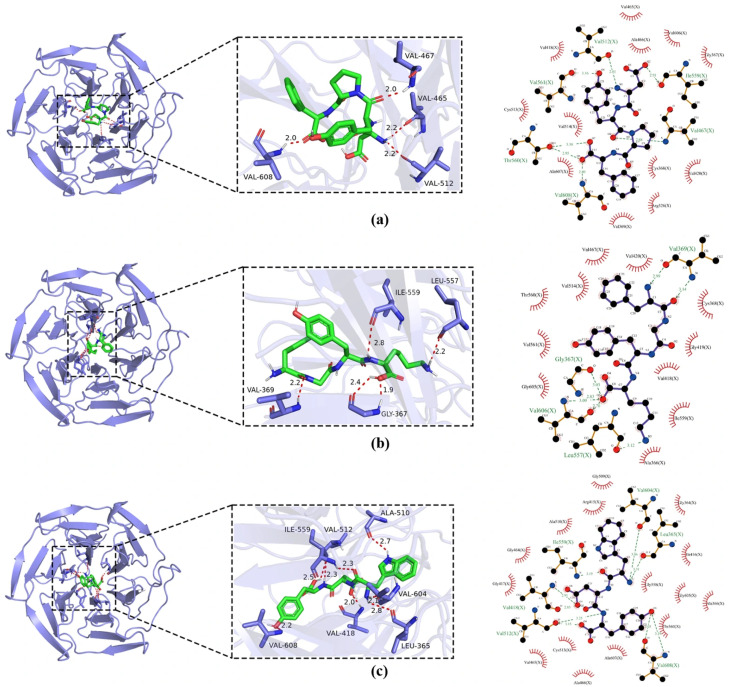
Molecular docking results of peptides. (**a**) DYPF; (**b**) FGYK; and (**c**) WDY with the Keap1 protein.

The binding affinity, expressed as the calculated binding free energy (ΔG, kcal/mol), is a critical metric for evaluating docking stability. Generally, a more negative value indicates a stronger and more favorable binding interaction [41]. As summarized in Figure 7, the three novel peptides—DYPF, WDY, and FGYK—demonstrated robust binding to the Keap1 protein. In detail, DYPF (ΔG = −8.6 kcal/mol) formed four hydrogen bonds with key residues Val608, Val467, Val465, and Val512 within the Keap1 binding pocket. Additionally, it engaged in extensive hydrophobic interactions with twelve surrounding residues, notably Val418, Cys513, Val514, and Ala607, which likely contribute significantly to the binding stability. WDY (ΔG= −10.3 kcal/mol) exhibited the strongest predicted affinity; WDY established a more extensive hydrogen-bonding network, forming seven hydrogen bonds with residues including Val512, Ala510, Val604, and Val608. It was also involved in hydrophobic contacts with fifteen residues, such as Gly509, Arg415, and Gly417, suggesting a highly complementary fit within the binding site. FGYK (ΔG = −8.0 kcal/mol) formed four hydrogen bonds with Ile559, Leu557, Val369, and Gly367. Its binding was further stabilized by hydrophobic interactions with eleven residues, including Val467, Val514, and Thr560. The molecular docking simulations indicate that the identified antioxidant peptides DYPF, WDY, and FGYK can spontaneously bind to the Keap1 protein in silico. Their favorable binding energies and specific interactions lend support to the hypothesis that they could disrupt the Keap1-Nrf2 interaction. Consequently, the activation of the Keap1-Nrf2-ARE pathway emerges as a plausible, though experimentally unconfirmed, molecular mechanism potentially underlying their observed in vitro chemical antioxidant activity. Nevertheless, these computational predictions necessitate rigorous validation in future cellular studies, including monitoring Nrf2 nuclear translocation, ARE-driven reporter gene expression, or quantifying downstream antioxidant enzyme activity under oxidative stress.

The molecular docking simulations strongly suggest that the identified antioxidant peptides DYPF, WDY, and FGYK can spontaneously and stably bind to the Keap1 protein. The favorable binding energies and specific interactions with key residues in the Keap1 binding pocket support the hypothesis that these peptides may act as potential disruptors of the Keap1-Nrf2 interaction. This provides a plausible molecular-level mechanism for their observed in vitro antioxidant activity, positing that they might activate the endogenous Nrf2-mediated antioxidant defense pathway. These computational findings warrant further experimental validation through cellular or biochemical assays.

## 4. Conclusions

This study successfully demonstrates the feasibility of converting Xuefeng black-bone chicken bone (BCB), a major processing by-product, into a valuable source of antioxidant peptides through a systematic bioprocess. The enzymatic hydrolysis conditions were effectively optimized using RSM, establishing a reliable model for producing hydrolysates with a high hydrolysis efficiency and potent antioxidant activity. Sequential purification via ultrafiltration and gel filtration chromatography conclusively confirmed that the low-molecular-weight fraction (<3 kDa) contained the most active components, aligning with the established structure–activity relationship of bioactive peptides. A significant outcome of this research was the identification of 21 peptide sequences from the BCB hydrolysate using LC–MS/MS. Through a rigorous in silico screening pipeline, three peptides (DYPF, WDY, and FGYK) were selected and chemically synthesized. Their experimentally validated in vitro antioxidant activities substantiated the predictions. Furthermore, molecular docking analysis offered a mechanistic hypothesis, indicating that these peptides could stably interact with the Keap1 protein in silico. This interaction suggests a potential mode of action that might involve disrupting the Keap1–Nrf2 interaction and subsequently potentiating cellular antioxidant defenses; however, this premise remains a hypothesis awaiting confirmation in biological systems. In summary, this work presents a comprehensive “from process to peptide” approach, encompassing optimization, purification, identification, synthesis, validation, and preliminary mechanistic exploration. The findings not only contribute to the fundamental knowledge of avian collagen-derived bioactive peptides but also offer a practical and sustainable strategy for the valorization of poultry by-products. The identified antioxidant peptides (DYPF, WDY, and FGYK) show a promising potential for development as natural antioxidants in the food, health, and cosmetic industries. Future research should focus on evaluating their stability, bioavailability, cellular uptake, and in vivo efficacy in model systems.

## Figures and Tables

**Table 1:** Response surface design and experimental results.

Group	Temperature (°C)	Time (h)	pH	DH (%)	ABTS Radical Scavenging Activity (%)
1	55	4	11	21.1197	71.417
2	50	3	9	19.7290	71.6475
3	55	3	10	23.7834	70.7512
4	50	3	11	21.7546	72.9725
5	50	4	10	24.4843	83.1871
6	50	5	11	23.7682	76.5597
7	55	4	9	21.8757	69.2358
8	50	4	10	24.7646	84.6855
9	50	4	10	24.7335	83.9669
10	55	5	10	23.3784	73.9548
11	50	4	10	25.6992	84.8116
12	45	4	11	21.9102	71.333
13	45	4	9	16.9725	63.0855
14	50	5	9	19.5812	71.0306
15	45	3	10	21.8676	69.9218
16	45	5	10	22.8956	73.7089
17	50	4	10	24.7646	84.5155

**Table 2:** Analysis of variance (ANOVA) for the second-order polynomial model that describes the DH.

Source	Sum of Squares	df	Mean Square	F-Value	*p*-Value	Significance
Model	81.98	9	9.11	32.68	<0.0001	**
A	5.30	1	5.30	19.01	0.0033	**
B	0.7743	1	0.7743	2.78	0.1395	
C	13.51	1	13.51	48.45	0.0002	**
AB	0.5134	1	0.5134	1.84	0.2169	
AC	8.10	1	8.10	29.07	0.0010	**
BC	1.17	1	1.17	4.19	0.0799	
A^2^	7.37	1	7.37	26.45	0.0013	**
B^2^	1.44	1	1.44	5.16	0.0573	
C^2^	40.37	1	40.37	144.82	<0.0001	**
Residual	1.95	7	0.2787			
Lack of fit	1.08	3	0.3586	1.64	0.3150	NS
Pure error	0.8753	4	0.2188			
Cor total	83.93	16				
R^2^ = 0.9768 R^2^_Adj_ = 0.9469						

Note: A, B, and C are temperature, hydrolysis time, and pH, respectively. ** *p* < 0.01, NS: not significant.

**Table 3:** Analysis of variance (ANOVA) for the second-order polynomial model that describes the ABTS radical scavenging activity.

Source	Sum of Squares	df	Mean Square	F-Value	*p*-Value	Significance
Model	700.74	9	77.86	62.44	<0.0001	**
A	6.68	1	6.68	5.36	0.0538	
B	12.40	1	12.40	9.95	0.0161	*
C	37.34	1	37.34	29.94	0.0009	**
AB	0.0851	1	0.0851	0.0683	0.8014	
AC	9.20	1	9.20	7.38	0.0299	*
BC	4.42	1	4.42	3.54	0.1018	
A^2^	284.29	1	284.29	227.99	<0.0001	**
B^2^	65.10	1	65.10	52.21	0.0002	**
C^2^	221.23	1	221.23	177.41	<0.0001	**
Residual	8.73	7	1.25			
Lack of fit	6.94	3	2.31	5.19	0.0728	NS
Pure error	1.78	4	0.4460			
Cor total	709.49	16				
R^2^ = 0.9877 R_Adj_ = 0.9719						

Note: A, B, and C are temperature, hydrolysis time, and pH, respectively. * *p* < 0.05, ** *p* < 0.01, NS: not significant.

**Table 4:** Quantity and proportion of F3 components by molecular weight.

MW (kDa)	Peptides Identified	Ratio (%)
<1	6429	99.09
1–3	57	0.88
>3	2	0.03

**Table 5:** Physicochemical properties and in silico bioactivity scores of the 21 novel antioxidant peptides from the F3 fraction of black-bone chicken bone hydrolysate.

Peptides	MW (Da)	Ip	Net Charge	Hydrophobic (kcal/mol)	Peptide Ranker	Wate Soluble	PFRS Score
GPGPW	512.24	5.55	0	8.39	0.9775	Poor	0.5623
FGGP	376.17	5.50	0	8.63	0.9678	Poor	0.5134
LYPF	538.28	5.50	0	4.37	0.9585	Poor	0.5477
NGPW	472.21	5.37	0	7.95	0.9553	Poor	0.5322
DYPF	540.22	2.95	−1	9.26	0.9371	Good	0.5282
WLGG	431.23	5.7	0	6.86	0.9284	Poor	0.5052
YFPH	562.25	7.65	0	7.95	0.8969	Poor	0.6026
FVHW	587.28	7.72	0	5.97	0.8934	Poor	0.5510
TPW	402.19	5.33	0	6.20	0.8790	Poor	0.5061
VGGW	417.20	5.58	0	7.65	0.8656	Poor	0.5060
FHY	465.20	7.60	0	7.81	0.8636	Poor	0.5765
WDY	482.18	3.18	−1	8.74	0.8611	Good	0.5345
SHYF	552.23	7.64	0	8.27	0.8293	Poor	0.5629
FLGH	472.24	7.69	0	8.42	0.8253	Poor	0.5173
TGYF	486.21	5.31	0	6.88	0.8092	Poor	0.5081
HLFY	578.28	7.60	0	6.56	0.8032	Poor	0.5178
FGYK	513.26	9.48	+1	9.43	0.7878	Good	0.5019
HIFY	578.28	7.6	0	6.69	0.7644	Poor	0.5062
GYL	351.18	5.58	0	7.09	0.7604	Poor	0.5084
HLHF	552.28	7.83	0	9.60	0.7442	Poor	0.5287
TWY	468.22	5.29	0	5.35	0.7249	Poor	0.5551

**Table 6:** Validation of in vitro antioxidant capacities of synthetic peptides.

Sequence	Molecular Weight (Da)	DPPH (%)	OH (%)
VC		93.49 ± 1.02 ^a^	99.50 ± 0.04 ^a^
WDY	540.56	28.10 ± 2.57 ^bc^	34.51 ± 0.70 ^d^
DYPF	483.49	31.06 ± 1.88 ^c^	36.78 ± 1.12 ^c^
FGYK	513.59	33.99 ± 1.55 ^b^	50.41 ± 0.78 ^b^

Note: Data are expressed as mean ± SD (*n* = 3). Different lowercase letters in the same column indicate significant differences (*p* < 0.05).

## Data Availability

The original contributions presented in this study are included in the article; further inquiries can be directed to the corresponding author.

## Supplementary Materials

The following supporting information can be downloaded at: https://www.mdpi.com/article/10.3390/foods15050942/s1. Figure S1. (a) ABTS radical scavenging activity, (b) hydroxyl radical scavenging activity, (c) DPPH radical scavenging activity, and (d) reducing power of enzymatic hydrolysates from non-hydrolyzed black chicken bone with varying molecular weights. Lowercase letters denote significant differences in antioxidant activity among peptide segments of the same molecular weight but different concentrations (*p* < 0.05). Uppercase letters indicate significant differences among different molecular weights at the same concentration (*p* < 0.05).
