# Supplementary material for: From Waste to Bioactive Ingredient: Integrated Extraction, Identification, and Validation of Novel Antioxidant Peptides from Xuefeng Black-Bone Chicken Bones
Source: Foods. 2026 Mar 7;15(5):942. doi: 10.3390/foods15050942 (PMC12984693; doi:10.3390/foods15050942)
Supplement: Supplementary file 1 [file foods-15-00942-s001.zip › Figure S1.pdf]

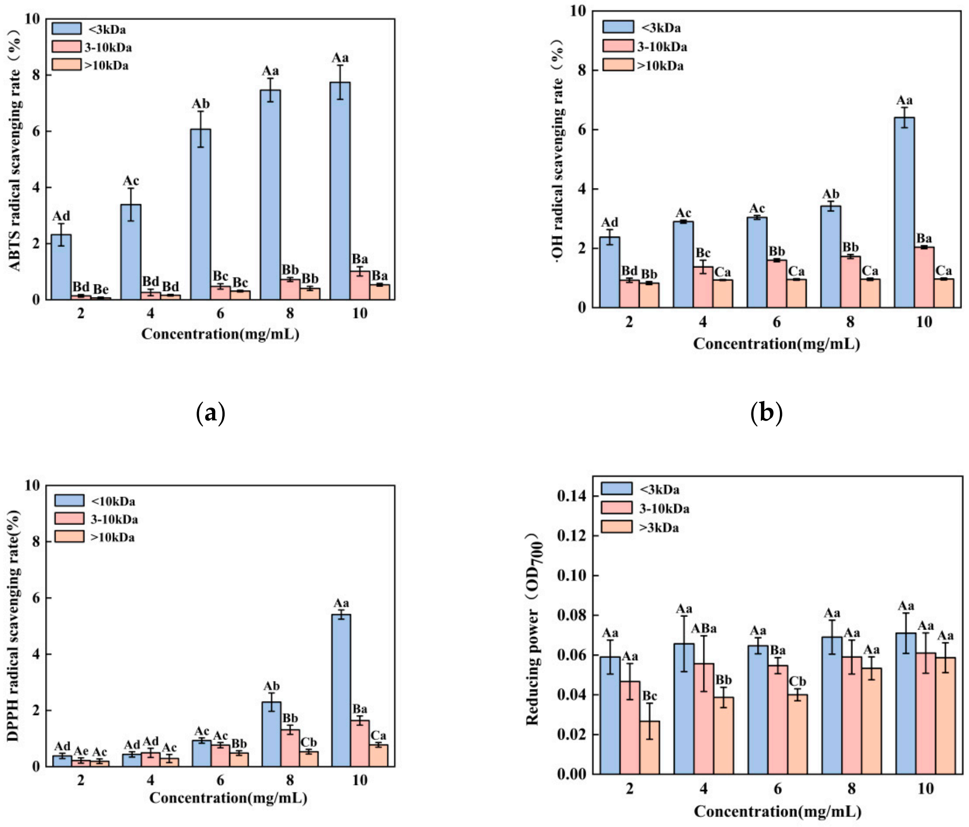

(c) (d)

Figure S1. (a)ABTS radical scavenging activity,(b) hydroxyl radical scavenging activity,(c)DPPH radical scavenging activity,and (d)reducing power of enzymatic hydrolysates from non-hydrolyzed black chicken bone with varying molecular weights. Lowercase letters denote significant differences in antioxidant activity among peptide segments of the same molecular weight but different concentrations ($P<0.05$). Uppercase letters indicate significant differences among different molecular weights at the same concentration ($P<0.05$).
